# Prognostic value of microvessel density in stage II and III colon cancer patients: a retrospective cohort study

**DOI:** 10.1186/s12876-019-1063-4

**Published:** 2019-08-16

**Authors:** Sjoerd H. den Uil, Evert van den Broek, Veerle M. H. Coupé, Thomas T. Vellinga, Pien M. Delis-van Diemen, Herman Bril, Eric J. Th. Belt, Onno Kranenburg, Hein B. A. C. Stockmann, Jeroen A. M. Belien, Gerrit A. Meijer, Remond J. A. Fijneman

**Affiliations:** 1 Department of Surgery, Spaarne Gasthuis, Boerhaavelaan 22, Haarlem, 2035 RC The Netherlands; 2 Department of Pathology and Medical Biology, University of Groningen, University Medical Center Groningen, Groningen, 9700 RB The Netherlands; 3 Department of Epidemiology and Biostatistics, VU University Medical Center, de Boelelaan 1089a, Amsterdam, 1081 HV The Netherlands; 4 Department of Surgical Oncology, UMC Utrecht Cancer Center, University Medical Center Utrecht, Heidelberglaan 100, Utrecht, 3584 CX The Netherlands; 5 Department of Pathology, The Netherlands Cancer Institute, Plesmanlaan 121, Amsterdam, 1066 CX The Netherlands; 6 Department of Pathology, Spaarne Gasthuis, Boerhaavelaan 22, Haarlem, 2035 RC The Netherlands; 7 Department of Surgery, Albert Schweitzer Ziekenhuis, Dordrecht, 3300 AK The Netherlands; 8 Department of Pathology, Amsterdam University Medical Centre, Vrije Universiteit Amsterdam, de Boelelaan 1117, Amsterdam, 1081 HV The Netherlands

**Keywords:** Colon cancer, Microvessel density, Angiogenesis, Prognosis

## Abstract

**Background:**

Microvessel density (MVD), as a derived marker for angiogenesis, has been associated with poor outcome in several types of cancer. This study aimed to evaluate the prognostic value of MVD in stage II and III colon cancer and its relation to tumour-stroma-percentage (TSP) and expression of HIF1A and VEGFA.

**Methods:**

Formalin-fixed paraffin-embedded (FFPE) colon cancer tissues were collected from 53 stage II and 54 (5-fluorouracil-treated) stage III patients. MVD was scored by digital morphometric analysis of CD31-stained whole tumour sections. TSP was scored using haematoxylin-eosin stained slides. Protein expression of HIF1A and VEGFA was determined by immunohistochemical evaluation of tissue microarrays.

**Results:**

Median MVD was higher in stage III compared to stage II colon cancers (11.1% versus 5.6% CD31-positive tissue area, *p* < 0.001). High MVD in stage II patients tended to be associated with poor disease free survival (DFS) in univariate analysis (*p* = 0.056). In contrast, high MVD in 5FU-treated stage III patients was associated with better DFS (*p* = 0.006). Prognostic value for MVD was observed in multivariate analyses for both cancer stages.

**Conclusions:**

MVD is an independent prognostic factor associated with poor DFS in stage II colon cancer patients, and with better DFS in stage III colon cancer patients treated with adjuvant chemotherapy.

**Electronic supplementary material:**

The online version of this article (10.1186/s12876-019-1063-4) contains supplementary material, which is available to authorized users.

## Background

The physiological role of angiogenesis at adult age is confined to wound- and bone healing and the female reproductive cycle, and therefore is activated temporarily. In contrast, the angiogenic pathway is often constitutively activated in tumours to meet their needs for nutrients and to facilitate tumour growth and metastasis. This is an early event in the development of cancer and can already be observed in pre-malignant lesions [1–3]. However, these newly formed blood vessels have a less distinct organized hierarchy [4], and are prone to vascular leakage for their irregular endothelial layer with intermediate spaces [5]. Interstitial blood pressure is increased leading to compromised blood flow and thus hypoxia and acidosis [6, 7]*.* Expression of the alpha subunit of hypoxia-inducible factor 1 (HIF1A) is stabilized as a result of this hypoxia and evokes angiogenesis by the upregulation of expression of vascular endothelial growth factor A (VEGFA) [8, 9]. This process is enhanced by the influence of for instance tumour associated macrophages, cancer-associated fibroblasts, and the extracellular matrix [10]. Constitutive upregulation of HIF1A and VEGFA expression can also be induced by oncogene signalling, e.g. by transforming growth factor-β (TFG-β), the involvement of the Wnt/β-catenin pathway and inhibition by the p53 pathway [11–16]. HIF1a is furthermore known to interact with apoptotic markers like Bax and Bcl-xL [17]. It leads to enhanced proliferation, survival and migration of endothelial cells, increased vascular permeability and altered gene expression [14, 18, 19]*.*

Desmoplastic tumour stroma interacts with and supports the tumour parenchyma, forming a microenvironment in which the tumour can progress. The tumour stroma and the microenvironment promote angiogenesis and tumour progression and eventually metastasis [10, 20]. There is increasing evidence that the proportion of this stroma in colon cancer is inversely related to survival [21, 22]. Consequently, tumours with high tumour-stroma percentage (TSP) are likely to express more angiogenic factors, leading to more angiogenesis, and angiogenesis-rich tumours may be associated with a worse prognosis.

In contrast to stroma percentage, there is no direct measure or single marker for angiogenesis to which survival can be correlated [21]. Microvessel density (MVD) has been analysed since the early 90’s as an angiogenesis-derived marker [23, 24]. The hypothesis that high-MVD tumours are associated with poor prognosis was indeed proven in breast cancer, where higher MVD was associated with poor survival [25, 26], and in non-small cell lung cancer where high MVD was associated with poor survival after surgery [27]*.* In colorectal cancer (CRC), prognostic value of MVD has remained inconclusive, although some publications suggest associations with survival [28–33].

The aim of this study was to examine the relation of MVD to disease-recurrence, in both stage II and stage III colon cancer patients, while taking into account the amount of tumour stroma (TSP) and expression of HIF1A and VEGFA.

## Materials and methods

### Study design and population

Based on a previously established well-documented retrospective cohort of 386 stage II and III colon cancer patients with no prior history of CRC [34], we here selected a subset of 53 stage II and 54 stage III colon cancer patients of whom whole tissue sections were available for MVD analysis. In this subset all stage III patients were treated with adjuvant 5-FU based chemotherapy, whilst all stage II patients were treated with surgical resection only. The tumours from these patients were microsatellite stable (MSS), as previously determined by PCR analysis [34]. Clinical data and tumour tissue was obtained conform the “Code for Proper Secondary Use of Human Tissue in The Netherlands” [35]. Baseline characteristics and clinicopathological data are shown in Additional file 2: Table S1.

### CD31 immunohistochemistry and microvessel density analysis

Four micrometer FFPE whole tissue sections were mounted on glass slides, deparaffinised and rehydrated. To identify (micro) vessels, sections were stained with a mouse monoclonal antibody directed against CD31 (anti human CD31, clone JC70A, catalogue number M0823, Dako, Heverlee, Belgium) in a 1/50 solution and using a Tris (pH 9) buffer for maximum retrieval in a microwave for 1 h. A Powervision+ method (Immunologic, Duiven, The Netherlands) was used as secondary antigens, after 1 h incubation at room temperature. These sections were digitized using a Mirax slide scanner system equipped with a 20x objective with a numerical aperture of 0,75 (3DHISTECH, Budapest, Hungary) and a Sony DFW-X710 Fire Wire 1/3″ type progressive SCAN IT CCD (pixel size 4,65 × 4,65 μm^2^) resulting in an actual scan resolution at 20x of 0,23 μm. Monitors used for selection and scoring were calibrated using Spyder2PRO software (v.1.0–16; Panone Colorvision, Regensdorf, Switzerland). Representative tumour tissue was delineated using Pannoramic Viewer (v 1.15.3, 3DHISTECH Ltd), and damaged parts and/or absence of tissue in delineated tumours were annotated as well, and later digitally excluded for analysis. A pathologist (HB) approved the delineating process. The delineated areas were exported as high resolution TIFF-files. This resulted in TIFF files of 60 to 320 Mb with a minimum resolution of approximately 2500 × 3100 pixels, depending on the original tumour size. A Java-based image processing program, ImageJ (v1.47, Wayne Rasband, 64bits), was used to import the TIFF files and to perform morphometric image analysis to detect and measure the microvessels. This full script, is a CD31-specific version of previously published work [36], see Additional file 1. In brief, this script analyses all stained pixels in included tissue in the delineated TIFF-files. Based on RGB colour codes, the CD31 positive cells were identified. The brown (clustered) pixels represented endothelial cells, whereas the non-brown pixel represented normal stroma, epithelial cells etc. A size-threshold of minimal hundred CD31-positive pixels was used as minimal size of microvessels. The total percentage of CD31-positive (clustered) pixels per tissue area analysed is used as measure for the average microvessel density in that whole tumour section.

### Tumour-stroma percentage

For TSP analysis, 4 μm FFPE tissue sections were mounted on glass slides, deparaffinised and rehydrated, and stained with haematoxylin-eosin (HE). Neoplastic epithelium and stroma was quantified using QProdit (Leica) stereology software. The borders of the tumour in each section were annotated. Subsequently, the software generated a 400-point grid for a 20x objective within these borders. At each point, the tissue was scored for epithelium or stroma. Tumour-stroma percentage was calculated using the number of stromal hits divided by the total number of both epithelial and stromal hits. Since some sections were damaged, 99 patients remained for analysis.

### HIF1A and VEGFA immunohistochemistry and TMA analysis

Tissue micro arrays (TMA) were generated from these patients as described previously [37]. Expression of HIF1A and VEGFA was determined by immunohistochemical evaluation of TMAs, using previously established workflows [37]. For HIF1A, antigen retrieval was performed in antigen retrieval solution (DAKO, Glostrup, Denmark) for 45 min at 96 °C. Then, the primary antibody against HIF1A (mouse monoclonal, clone 54, catalogue number 610958, BD, Franklin Lakes, USA) was incubated for 30 min with a 1/500 dilution at room temperature. The amplification reagent from the Catalazyd Signal Amplification system (CSA, Dako kit) was used for detection of the staining. For VEGFA expression (mouse monoclonal, clone VG1, catalogue number MS-1467-P1, Neomarkers, Fermont, USA) a Tris (pH 9) buffer in the microwave was used for antigen retrieval, with subsequent incubation for 1 h and secondary visualization also by a Powervision+ method.

Protein expression analysis of TMAs was performed as described previously [37]. In brief, six cores per patient were examined and scored blindly for intensity (negative, weak, moderate and strong) of stained cells, using dedicated TMA scoring software (Pannoramic Viewer, v1.15.5; 3DHISTECH Ltd). Scores were internally corrected for stromal- and background staining. Damaged and missing cores were not scored. Expression was scored in cytoplasm of epithelial cells. Scores were obtained for 103 (HIF1A) and 100 (VEGFA) patients, respectively. For further statistical analyses, all scores were converted to dichotomous values using ROC-based cross-validation analysis [37].

### Statistical analysis

Kolmogorov-Smirnov (KS) test was used to assess the normality of the distribution of MVD- and TS*P*-values. For analysis of differences in clinical and histological baseline parameters between study groups (stage and MVD) independent-t-testing, Mann-Whitney U and chi-square tests were used. In both stage II and III, continuous MVD- and stromal data was dichotomized for further (survival) analysis, based on highest specificity and sensitivity in ROC-analysis. This resulted in high-MVD when MVD was higher than 5.45% for stage II, and higher than 8.91% for stage III colon cancer. Stroma was subsequently defined as high if TSP > 43,1% for stage II, and TSP > 49,2% for stage III colon cancer. Difference in disease free survival (DFS) was visualised with Kaplan Meier curves and log-rank. Hazard ratio’s (HR) and 95% confidence interval (95% CI) were estimated with cox-regression analysis. Multivariate analysis was performed using stepwise backwards Cox regression, with DFS as dependent variable (p-out = 0.1). Similar statistics were performed on expression scores of HIF1A and VEGFA. Associations between MVD, TSP, HIF1A and VEGFA were analysed with chi-square tests and spearman’s rho-test. All statistical analyses were processed in SPSS (IBM SPSS Statistics for windows, SPSS Inc., Chicago, Illinois, USA), with two-sided analysis and a significance level of *p* < 0.05.

## Results

### MVD in stage II and stage III colon cancer

**Fig. 1:**
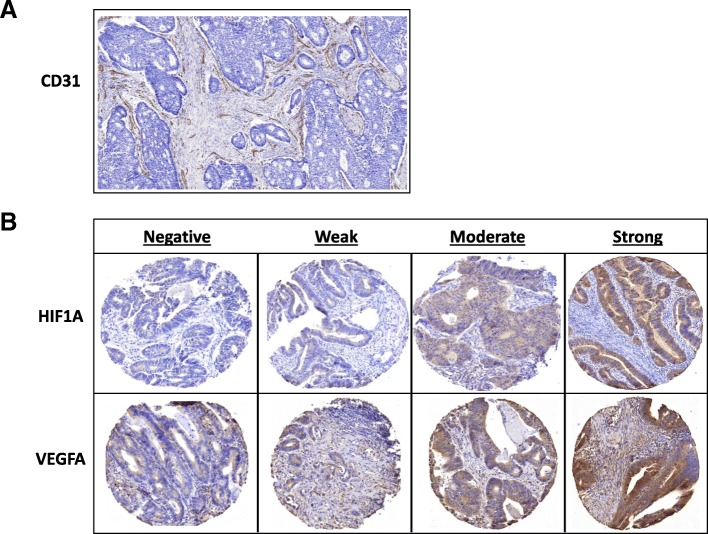
Examples of immunohistochemical stainings for **a**: CD31 on whole tissue section; and **b**: HIF1A and VEGFA on TMA cores, scored as negative, weak, moderate, strong

For determining MVD, whole tissue sections were stained with CD31 (Fig. 1a). Baseline clinical and pathological data characteristics of the cohort are presented in Additional file 2: Table S1, stratified for stage II and III colon cancer. Besides differences that are inherent to a comparison of stage II to stage III patients (adjuvant chemotherapy, T- and N-stadium), stage III patients were significantly younger (65,5 versus 72.7 years; *p* = 0.004) and had significantly more angioinvasion, defined by the observation of epithelial cells within the (lymph) vascular lumen (38.9% versus 11.3%, *p* = 0.001).

**Fig. 2:**
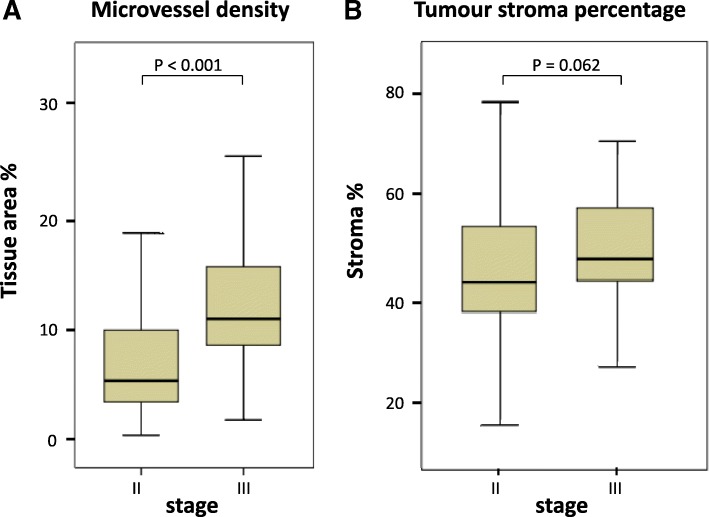
Comparison between stage II and stage III colon cancer patients for **a** microvessel density; and **b** tumour-stroma percentage. *P* values were obtained by Mann-Whitney U analysis

Mean MVD of 107 tumours was 10.4%, with a median of 9.0. In stage III tumours, MVD was significantly higher compared to stage II (11.1 and 5.6%, respectively; *p* < 0.001, Fig. 2a). Within stage II, no differences were seen in clinicopathological characteristics between MVD-high and –low group. In stage III tumours, only more ulceration (*p* = 0.042) and less recurrences in high-MVD patients (*p* = 0.013) were observed. Clinicopathological characteristics for high versus low MVD, within both stages, are presented in Table 1.

**Table 1:** Clinicopathological characteristics

	Stage II: *n* = 53 (%)			Stage III: *n* = 54 (%)		
	MVD-low *n* = 26 (%)	MVD-high *n* = 27 (%)	*P*-value	MVD-low *n* = 15 (%)	MVD-high *n* = 39 (%)	*P*-value
Sex						
Male	14 (53.8)	14 (51.8)		10 (66.7)	25 (64.1)	
Female	12 (46.2)	13 (48.2)	0.88	5 (33.3)	14 (35.9)	0.86
Age, mean (s.d.) in years	73.7 (11.3)	70.9 (13.3)	0.41	63.6 (9.8)	66.6 (10.1)	0.32
Right sided tumour	9 (34.6)	8 (29.6)	0.70	7 (46.7)	18 (46.2)	0.97
Tumour diameter, mean (s.d.) in mm	39.2 (20.7)	40.80 (19.3)	0.61	36.4 (10.5)	33.9 (13.4)	0.44
Histological grade						
Good	2 (7.7)	3 (11.1)		1 (6.7)	1 (2.6)	
Average	23 (88.5)	23 (85.2)		12 (80.0)	35 (89.7)	
Poor	1 (3.8)	1 (3.7)	0.91	2 (13.3)	3 (7.7)	0.61
Tumour stage						
T1	–			–	1 (2.6)	
T2	–			1 (6.7)	6 (15.4)	
T3	23 (88.5)	26 (96.3)		10 (66.7)	30 (76.9)	
T4	3 (11.5)	1 (3.7)	0.28	4 (26.7)	2 (5.1)	0.13
Nodal stage (stage III)						
N1	–	–		12 (80.0)	22 (56.4)	
N2	–	–	–	3 (20.0)	17 (43.6)	0.11
Mucinous differentiation.	8 (30.8)	4 (14.8)	0.17	1 (6.7)	4 (10.3)	0.68
Ulceration	18 (69.2)	23 (85.2)	0.17	10 (66.7)	35 (89.7)	0.042^*^
Angioinvasion	2 (7.7)	4 (14.8)	0.41	7 (46.7)	14 (35.9)	0.47
Perforation						
No	24 (92.3)	24 (88.9)		14 (93.3)	37 (94.9)	
Before surgery	1 (3.8)	1 (3.7)		1 (6.7)	1 (2.6)	
During surgery	–	1 (3.7)		–	–	
After surgery	1 (3.8)	1 (3.7)	0.81	–	1 (2.6)	0.64
Tumour spill	1 (3.8)	2 (7.4)	0.58	1 (6.7)	–	0.10
Adjuvant chemo	0	0	–	15 (100.0)	39 (100.0)	–
Recurrence	6 (23.1)	12 (44.4)	0.10	11 (73.3)	14 (35.9)	0.013^*^
CRC mortality	6 (23.1)	9 (33.3)	0.41	8 (53.3)	12 (30.8)	0.12
Overall mortality	14 (53.8)	16 (59.3)	0.69	9 (60.0)	17 (43.6)	0.28
Follow up, mean (s.d.) in months	70.5 (32.6)	58.1 (35.9)	0.19	52.3 (33.7)	57.7 (27.4)	0.54

Baseline characteristics and clinicopathological data stratified for low and high MVD for both stage II and III colon cancer. *P*-values were calculated by chi-square test or independent t-testing for continuous data

*Significant *p*-value < 0.05

### High MVD is associated with poor DFS in stage II, and better DFS in adjuvant treated stage III colon cancers

**Fig. 3:**
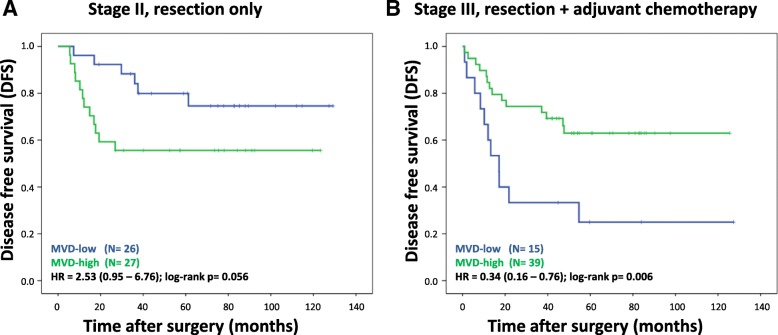
Kaplan Meier curves for DFS of stage II (**a**) and stage III (**b**) colon cancer patients, stratified for high and low MVD. Hazard ratio (HR), 95% confidence interval, and log-rank *p*-values are reported

To investigate the univariate association of MVD with survival in stage II patients, DFS was analysed and visualized using Kaplan Meier curves. For stage II, high MVD tended to be associated with worse DFS (HR 2.53; 95% CI 0.95–6.76; Log-rank *p* = 0.056, Fig. 3a). Similarly, the univariate effect of MVD on DFS in stage III was investigated. In contrast to the association in stage II, high MVD is associated with better DFS in stage III patients, all treated with adjuvant chemotherapy (HR 0.34; 95% CI 0.16–0.76; Log-rank *p* = 0.006, Fig. 3b).

### No association between DFS and TSP, HIF1A or VEGFA

Examples of expression of HIF1A and VEGFA are shown in Fig. 1b, and results and survival curves for TSP, HIF1A and VEGFA are presented in Additional file 3: Figure S1-S3. In contrast to MVD, TSP was not significantly different between stage II and III colon cancers (*p* = 0.062, Fig. 2b). More stroma showed a trend of being related to poor DFS in stage II (HR 2.07; 95% CI 0.76–5.60; Log-rank *p* = 0.144, Additional file 3: Figure S1a). Also for expression of HIF1A and VEGFA, no statistically significant associations were observed (Additional file 3: Figure S2-S3).

### Microvessel density is an independent prognostic factor in stage II and III colon cancer patients

A multivariate model for 5-year DFS was built using stepwise backward Cox-regression. MVD, TSP, HIF1A, VEGFA, right-sided, diameter, degree of differentiation, ulceration and angioinvasion were included for analysis. In both stage II and stage III colon cancer, MVD was retained in the model significantly, demonstrating its added value as a prognostic biomarker in a multivariate setting.

In addition to MVD, only right-sidedness and ulceration were retained in stage II colon cancer. For stage III colon cancer ulceration and angioinvasion in stage III were retained in the model (Table 2). TSP, HIF1A and VEGFA were not associated with DFS in this multivariate assay. No correlations between MVD and TSP or expression of HIF1A and VEGFA were found. MVD was correlated to expression of VEGFA in stage II colon cancer (correlation coefficient − 0.331, *p* = 0.020, Additional file 2: Table S2).

**Table 2:** Results for multivariate analysis

Stage	Parameters	HR	95%-CI	*P*-value
II	Right-sided tumour	0.18	0.04–0.83	0.027
	Ulceration	6.80	0.89–52.23	0.065
	High microvessel density	4.50	1.38–14.64	0.013^*^
III	Ulceration	0.32	0.10–1.06	0.062
	Angioinvasion	4.38	1.71–11.23	0.002
	High microvessel density	0.34	0.13–0.90	0.031^*^

Multivariate backward Cox-regression analysis for 5-year disease free survival of high microvessel density and clinicopathological parameters that were retained in the model, in stage II and III colon cancer patients. *HR* Hazard ratio, *95%-CI* 95% confidence interval

*Significant *p*-value < 0.05

## Discussion

This study addressed the analysis of microvessel density and its relation to disease stage and prognosis, in microsatellite stable stage II and III colon cancer patients. MVD was higher in stage III compared to stage II colon cancers. Previously it has been shown that MVD increases during the progression from normal mucosa, through adenomas to carcinomas [38]. It is plausible that during the evolution from stage I carcinoma to metastasized disease (stage IV), angiogenesis and MVD are enhanced to meet the increasing demands of tumour growth and progression.

In stage II, high MVD was related to worse disease outcome, i.e. worse DFS, in particular observed as a significant effect in the multivariate analysis. Thus, stage II colon cancers with high MVD might represent a biological subset of cancers with unfavourable characteristics and a tendency to progression, in line with the observation mentioned previously, leading to worse prognosis. A similar trend, albeit not reaching statistical significance, was found for TSP. All stage III tissues were obtained from patients who were treated with adjuvant chemotherapy after resection of their primary tumour. In contrast to stage II, high MVD was related to improved DFS in stage III patients. Stage III cancers already proved to have lymphatic potential, and can only progress to metastasized disease, stage IV. Although the primary tumours are already resected, residual tumour tissue, whether located in lymphatic tissue or already as subclinical distant metastasis, potentially exert the same tumour characteristics as the primary tumour [39]. Therefore, residual tumour tissue or early recurrent tumours, from primary tumours with high MVD, might have higher MVD as well and potentially allow better penetration for the adjuvant 5-FU based chemotherapy. This might explain the better prognosis of 5-FU treated stage III colon cancers with high MVD. It seems that even though high-MVD stage III cancers should have worse prognosis when untreated, they actually might predict better response to adjuvant chemotherapy. This hypothesis might also explain why Bevacizumab in stage II colon cancer does not improve DFS [40]. In stage II, with already fewer (micro) vessels present, only patients with high MVD (high risk) might benefit from inhibiting formation of new vessels. This might explain why there was no improvement of DFS for the entire group of stage II patients, since the group with favourable prognosis (low MVD) might show no further improvement by reduction of the already low vessel density. Potentially there is benefit from Bevacizumab in lower stages of colon cancer, though restricted to selected cases with high MVD.

Tumour-stroma percentage and the expression of HIF1A and VEGFA, although functionally interconnected, were not significantly associated with MVD in this study, except for correlation between MVD and expression of VEGFA in stage II. Interestingly, for the association of stromal percentage with DFS for stage II and III, an opposite effect on DFS was observed in both stages similar as for MVD.

With regards to the method of MVD-analysis, several measures were taken to avoid some well-known methodological difficulties. In literature concerning MVD in colon cancer, results on prognosis were ambiguous, possibly for its wide range of methods. Antibodies used to visualize endothelial cells differ amongst studies (CD31, CD34, factor VIII, miRNA-126) [28], and sampling of the measurement area within the tumour is another critical factor. It is accepted to define MVD in ‘hot-spots’, but there is no consensus about the number of hotspots needed to count [24, 29–31, 41]. Furthermore, ‘hotspot’ may refer to the invasive margin of the tumour, or the area in the tumour with highest MVD by ‘eyeballing’. Both selection methods may be observer-dependent. Finally, microvessels can be counted manually or digitized using quantitative image analysis, of which the latter has proven to have more accuracy and prognostic relevance [24]. These and other ambiguities may contribute to the fact that MVD is not unanimously described as a prognostic factor, prohibiting it from being implemented in standard histopathological examination. To avoid such observer-dependent area selection, MVD was analysed digitally and in whole sections in which the entire tumour-area was annotated. This excluded both the bias of hot-spot diameter/selection, as the disadvantages of manual counting. It contributed to a more robust, feasible, reproducible and observer-independent method. To identify endothelial cells, CD31 antibodies were used as a commonly accepted marker [27], taking into account that it can be found on platelets and white blood cells to some degree as well. On the other hand, it is more sensitive for younger and more immature vessels.

## Conclusions

MVD is a surrogate marker of angiogenesis in tumours, direct measurement of which so far has remained impossible. Still, measuring MVD remains subject to some practical challenges, of which some were tackled in this study. In the present study, an increased MVD was seen in stage III colon cancer patients, in comparison to stage II. MVD appeared to be an independent prognostic factor associated with poor DFS in stage II colon cancer patients, and with better DFS in stage III colon cancer patients who were treated with adjuvant 5-FU based chemotherapy afterwards. This latter observation may be of particular clinical interest, pending further validation.

## Additional files

Additional file 1: Containing the CD31 specific script for microvessel density analysis. (DOCX 23 kb)

Additional file 2: **Table S1.** The clinicopathlogical data stratified for stage II and III patients. **Table S2.** The results of the correlation analysis of MVD, TSP, HIF1a and VEGFa. (DOCX 18 kb)

Additional File 3: **Figure S1.** DFS stratified for high and low stromal percentage. **Figure S2.** DFS stratified for high and low expression of HIF1A. **Figure S3.** DFS stratified for high and low expression of VEGFA. (PPTX 101 kb)

## Data Availability

The datasets used and/or analysed during the current study are available from the corresponding author on reasonable request.

## Abbreviations

5-FU: 5-fluorouracil
CI: Confidence interval
DFS: Disease free survival
FFPE: Formalin-fixed paraffin-embedded
HR: Hazard ratio
MSI: Microsatellite instability
MSS: Microsatellite stable
MVD: Microvessel density
TMA: Tissue micro array
TSP: Tumour stroma percentage
