# Supplementary material for: Prognostic value of microvessel density in stage II and III colon cancer patients: a retrospective cohort study
Source: BMC Gastroenterol. 2019 Aug 16;19:146. doi: 10.1186/s12876-019-1063-4 (PMC6698008; doi:10.1186/s12876-019-1063-4)
Supplement: Supplementary file 1 — Containing the CD31 specific script for microvessel density analysis. (DOCX 23 kb) [file 12876_2019_1063_MOESM1_ESM.docx]

**Supplementary methods**

*Full script for ImageJ analysis of MVD using CD31-stained slides*

// macro count microvesseldensity on CD31 stained slides

// as well as count number of blue (Hematox) stained nuclei

// Author Jeroen A.M. Beliën

// date: 23-08 till 02-09-2010

// updated algorithm 20-01-2014 v4, included the run("Options...",...) and run("Colors...",...), and replaced run("Invert"); on original places

// included relativeprecentage area brown/blue

//

// This is the batch version as originally created 05-05-2014 by JAMB and translated to English 22-10-2018

//

// plugins needed:

// thresholdcolour: with RGBtoLab_.class/.java in plugin folder

// ij-ImageIO_.jar needed for run("Open ...")

// folder colour functions that contains Colour_Deconvolution.class/.java

// ij-plugins_toolkit.jar needed for multiband Sobel

// Multi_Thresholder.jar for MultiThresholder tool

var OriginalImage, LumenImage, MeasurementImage, ExclFromMeasurementImage, ExclFromEverythingImage;

var red_exclude_low_th, lumen_low_th;

var totarea_lumen,totarea_tissue;

var image_name;

requires("1.44e");

run("Options...", "iterations=1 black count=1");

run("Colors...", "foreground=white background=black selection=yellow");

dir1 = getDirectory("Choose Source Directory ");

dir2 = getDirectory("Choose Destination Directory ");

list1 = getFileList(dir1);

setBatchMode(true);

for (my_list1=0; my_list1<list1.length; my_list1++) {list = getFileList(dir1+list1[my_list1]);

showProgress(my_list1, list1.length+1);

if (list.length>0) {

overall_result_file_area_percentage = File.open(dir2+"Results_of_"+"_"+list[0]+"_till_"+list[list.length - 1]+".txt");

print(overall_result_file_area_percentage, "Imagename;Tot_area_brown;Tot_area_demarcacted_area;Percentage");

for (my_list=0; my_list<list.length; my_list++) {

run("Open ...", "image=["+dir1+list1[my_list1]+list[my_list]+"] image=["+dir1+list1[my_list1]+list[my_list]+"]");

image_name = File.name;

overall_result_file_vessels = dir2+"Results_of_"+image_name+"_microvessels.xls";

overall_result_file_nuclei = dir2+"Results_of_"+image_name+"_nuclei.xls";

OriginalImage = getImageID();

AssessCD31stained_microvesselsAndNuclei();

//close();

// close all to clean up

wlist = getList("window.titles");

if (wlist.length==0) {

// nothing to do

}

else {

for (i=0; i<wlist.length; i++) {

//print(" "+wlist[i]);

selectWindow(wlist[i]);

run("Close");

}

}

if (nImages==0) {

// nothing to do

}

else {

while (nImages>0) {

selectImage(nImages);

close();

}

}

}

File.close(overall_result_file_area_percentage);

}

}

function AssessCD31stained_microvesselsAndNuclei() {

// Parameters saved are:

// area as Area

// centroid as X Y

// center (center of mass) as XM YM

// perimeter as Perim.

// bounding (bounding rectangle) as BX BY Width Height

// fit (fit elipse) as Major Minor Angle

// shape (shape descriptors) as Circ. AR Round Solidity

red_exclude_low_th=190;

lumen_low_th=240;

// set scale so it will not use an old scaling

// depending on scalebar (and compression set when making snapshot or exporting) set it accordingly

// Default -> run("Set Scale...", "distance=0 known=0 pixel=1 unit=pixel global");

run("Set Scale...", "distance=108 known=200 pixel=1 unit=mu global");

// This image will hold the tissue on which measurements will be done

selectImage(OriginalImage);

run("Duplicate...", "title=Measurement_area");

MeasurementImage = getImageID();

// Get rid of red demarcated areas but keep the orange part

selectImage(OriginalImage);

run("Duplicate...", "title=ExclFromMeasurement_area"); // orange part

ExclFromMeasurementImage = getImageID();

selectImage(OriginalImage);

run("Duplicate...", "title=ExclFromEverything"); // red part

ExclFromEverythingImage = getImageID();

// select red annotations

selectImage(ExclFromEverythingImage);

min=newArray(3);

max=newArray(3);

filter=newArray(3);

a=getTitle();

run("RGBtoLab ");

run("RGB Stack");

run("Convert Stack to Images");

selectWindow("Red");

rename("0");

selectWindow("Green");

rename("1");

selectWindow("Blue");

rename("2");

min[0]=0;

max[0]=255;

filter[0]="pass";

min[1]=red_exclude_low_th;

max[1]=255;

filter[1]="pass";

min[2]=0;

max[2]=255;

filter[2]="pass";

for (i=0;i<3;i++){

selectWindow(""+i);

setThreshold(min[i], max[i]);

run("Make Binary", "thresholded remaining");

if (filter[i]=="stop") {

run("Invert");

}

}

imageCalculator("AND create", "0","1");

imageCalculator("AND create", "Result of 0","2");

for (i=0;i<3;i++){

selectWindow(""+i);

close();

}

selectWindow("Result of 0");

close();

selectWindow("Result of Result of 0");

rename(a);

// Colour Thresholding------------

run("8-bit");

//setThreshold(10, 109);

//run("Convert to Mask");

run("Fill Holes"); // Detected area inside red demarcated area has value 255

run("Invert"); // So invert (not invert LUT): this area should be excluded from all measurements

// Now threshold lumen

selectImage(OriginalImage);

run("Duplicate...", "title=lumen_area");

LumenImage = getImageID();

selectImage(LumenImage);

a=getTitle();

// Colour Thresholding------------

run("8-bit");

setThreshold(lumen_low_th,255);

run("Convert to Mask");

run("Invert"); // So invert (not invert LUT): Needed to exclude this area from annotations with respect to calculations;

// Also remove area that needs to be excluded from the lumen area

imageCalculator("AND","lumen_area", "ExclFromEverything");

// Now obtain DAB positive objects

selectImage(OriginalImage);

run("Set Scale...", "distance=0 known=0 pixel=1 unit=pixel global");

run("Duplicate...", "title=[brown]");

// Obtain number of brown objects (microvessels)

run("Colour Deconvolution", "vectors=[H DAB]");

selectWindow("Colour Deconvolution");

close();

selectWindow("brown-(Colour_3)");

close();

selectWindow("brown-(Colour_2)");

run("Duplicate...", "title=[brown_sobel_input]");

selectWindow("brown-(Colour_2)");

setThreshold(1, 220);

run("Convert to Mask");

imageCalculator("AND","brown-(Colour_2)", "lumen_area");

imageCalculator("AND","brown-(Colour_2)", "ExclFromEverything");

run("Duplicate...", "title=[Brown_area]");

// get rid off possible wrong result by dialtion on upper and left part of image (probably edge effects)

selectWindow("brown-(Colour_2)");

makeRectangle(1, 1, getWidth(), getHeight());

run("Crop");

// seed image

run("Duplicate...", "title=[seed]");

//Make seed binary

selectWindow("seed");

run("8-bit");

setThreshold(1, 255); //create binary image

run("Convert to Mask");

// 04-02-2014 Take brown colour image as sobel input and not original image

selectWindow("brown_sobel_input");

run("Multiband Sobel edges");

setAutoThreshold("Default dark");

//setThreshold(100.7446, 1167.7217);

run("8-bit");

run("MultiThresholder", "Moments");

run("Convert to Mask");

run("Invert");

// get rid off possible wrong result by dialtion on upper and left part of image (probably edge effects)

makeRectangle(1, 1, getWidth(), getHeight());

run("Crop");

imageCalculator("AND create", "brown", "brown_sobel_input - Sobel edges"); // mask image

run("Duplicate...", "title=[mask]");

//Make mask binary

run("8-bit");

setThreshold(1, 255); //create binary image

run("Convert to Mask");

selectWindow("seed");

run("Duplicate...", "title=[orig_seed]");

selectWindow("seed");

run("Dilate");

imageCalculator("AND", "seed","mask");

selectWindow("seed");

imageCalculator("Subtract create", "seed", "orig_seed");

selectWindow("Result of seed");

getHistogram(values,counts,256);

threshold_counts = counts[255]/200;

if (threshold_counts < 50) {

threshold_counts = 50;

}

//print("aantal witte 255 pixels = "+ counts[255]);

selectWindow("Result of seed");

close();

selectWindow("orig_seed");

close();

while (counts[255] > threshold_counts) {

selectWindow("seed");

run("Duplicate...", "title=[orig_seed]");

selectWindow("seed");

run("Dilate");

imageCalculator("AND", "seed","mask");

selectWindow("seed");

imageCalculator("Subtract create", "seed", "orig_seed");

selectWindow("Result of seed");

getHistogram(values,counts,256);

//print("aantal witte 255 pixels = "+ counts[255]);

selectWindow("Result of seed");

close();

selectWindow("orig_seed");

close();

}

run("Set Measurements...", "area centroid center perimeter bounding fit shape limit redirect=None decimal=3");

selectWindow("seed");

run("Duplicate...", "title=[Brown_results]");

selectWindow("seed");

imageCalculator("AND","seed", "lumen_area");

imageCalculator("AND","seed", "ExclFromEverything");

run("Analyze Particles...", "size=100-Infinity circularity=0.00-1.00 show=Masks display clear");//size from 1000 back to 100 after attempt on analyzing 1:8 scaled image

//print("Number of brown objects corrected = "+nResults);

saveAs("Results", overall_result_file_vessels);

// Parameters returned:

// area as Area

// centorid as X Y

// center (center of mass) as XM YM

// perimeter as Perim.

// bounding (bounding rectangle) as BX BY Width Height

// fit (fit elipse) as Major Minor Angle

// shape (shape descriptors) as Circ. AR Round Solidity

// Obtain number of nuclei

// selectImage(OriginalImage);

// run("Colour Deconvolution", "vectors=[H DAB]");

selectWindow("brown-(Colour_1)");

run("Duplicate...", "title=[Blue_area]");

selectWindow("brown-(Colour_1)");

setAutoThreshold("Default");

setThreshold(0, 100);// 100 almost OK for blue component

run("Convert to Mask");

run("Set Measurements...", "area centroid center perimeter bounding fit shape limit redirect=None decimal=3");

imageCalculator("AND","brown-(Colour_1)", "lumen_area");

imageCalculator("AND","brown-(Colour_1)", "ExclFromEverything");

run("Analyze Particles...", "size=10-Infinity circularity=0.00-1.00 show=Masks display clear"); //reduced 100 size criterium to 10

//print("Number of blue objects uncorrected at threshold 100 = "+nResults);

saveAs("Results", overall_result_file_nuclei);

//Calculate blue and brown tissue area (so lumen and exclude are taken away!!!)

// Brown area is already thresholded at 220

total_brown_area=0;

selectWindow("Brown_area");

// Everything with value 255 (binary image) will be measured as object!

run("Set Measurements...", "area limit redirect=None decimal=3"); // General settings

run("Analyze Particles...", "size=0-Infinity circularity=0.00-1.00 show=Nothing clear");

for (i=0; i<nResults; i++) {

total_brown_area = total_brown_area + getResult("Area",i);

}

//print("Total area brown staining in mu = "+total_brown_area);

//print("Number of brown objects uncorrected at threshold 220 = "+nResults);

// Threshold blue image at 220

selectWindow("Blue_area");

setThreshold(0, 220);// 220 OK for blue area component

run("Convert to Mask");

run("Invert");

total_blue_area=0;

// Everything with value 255 (binary image) will be measured as object!

run("Set Measurements...", "area limit redirect=None decimal=3"); // General settings

run("Analyze Particles...", "size=0-Infinity circularity=0.00-1.00 show=Nothing clear");

for (i=0; i<nResults; i++) {

total_blue_area = total_blue_area + getResult("Area",i);

}

//print("Total area blue staining in mu = "+total_blue_area);

//print("Number of blue objects uncorrected at threshold 220 = "+nResults);

selectImage(LumenImage);

// lumen is already inverted so we obtain tissue/stroma component

// Everything with value 255 (binary image) will be measured as object!

run("Set Measurements...", "area limit redirect=None decimal=3"); // General settings

run("Analyze Particles...", "size=0-Infinity circularity=0.00-1.00 show=Nothing clear");

for (i=0; i<nResults; i++) {

total_demarcated_tissue_area = total_demarcated_tissue_area + getResult("Area",i);

}

//print("Total total_demarcated_tissue_area in mu = "+total_demarcated_tissue_area);

area_percentage = 0;

area_percentage = (total_brown_area/total_demarcated_tissue_area)*100;

//print("percentage brown/demarcated_area = "+area_percentage);

print(overall_result_file_area_percentage,dir1+list[my_list]+";"+total_brown_area+";"+total_demarcated_tissue_area+";"+area_percentage);

}
