# Supplementary material for: Prognostic value of microvessel density in stage II and III colon cancer patients: a retrospective cohort study
Source: BMC Gastroenterol. 2019 Aug 16;19:146. doi: 10.1186/s12876-019-1063-4 (PMC6698008; doi:10.1186/s12876-019-1063-4)
Supplement: Supplementary file 2 — Table S1. The clinicopathlogical data stratified for stage II and III patients. Table S2. The results of the correlation analysis of MVD, TSP, HIF1a and VEGFa. (DOCX 18 kb) [file 12876_2019_1063_MOESM2_ESM.docx]

**Supplementary Table 1**Baseline characteristics (n=107) and clinicopathological data stratified for stage II and stage III colon cancer. P-values were calculated by chi-square test, independent t-testing or Mann Whitney-U. P-values <0.05 are printed in bold.

| Clinicopathological characteristics | **Total:**  **n= 107 (%)** | **Stage II**  **n= 53 (%)** | **Stage III**  **n=54 (%)** | **p-value** |
| --- | --- | --- | --- | --- |
| Sex  Male  Female | 63 (58.9)  44 (41.1) | 28 52.8)  25 (47.2) | 35 (64.8)  19 (35.2) | 0.21 |
| Age, mean (s.d.) in years | 69.0 (11.7) | 72.7 (12.3) | 65.5 (10.0) | **0.004** |
| Right sided tumour | 42 (39.3) | 17 (32.1) | 25 (46.3) | 0.13 |
| Tumour diameter, mean (s.d.) in mm | 37.2 (16.7) | 40.0 (19.8) | 34.6 (12.7) | 0.41 |
| Histological grade  Good  Average  Poor | 7 (6.5)  93 (86.9)  7 (6.5) | 5 (9.4)  46 (86.8)  2 (3.8) | 2 (3.7)  47 (87.0)  5 (9.3) | 0.28 |
| Tumour stage  T1  T2  T3  T4 | 1 (0.9)  7 (6.5)  89 (83.2)  10 (9.3) | -  -  49 (92.5)  4 (7.5) | 1 (1.9)  7 (13.0)  40 (74.1)  6 (11.1) | **0.026** |
| Nodal stage (stage III)  N1  N2 | 34 (31.8)  20 (18.7) | -  - | 34 (63.0)  20 (37.0) | - |
| Mucinous differentiation. | 17 (15.9) | 12 (22.6) | 5 (9.3) | 0.058 |
| Ulceration | 86 (80.4) | 41 (77.4) | 45 (83.3) | 0.44 |
| Angioinvasion | 27 (25.2) | 6 (11.3) | 21 (38.9) | **0.001** |
| Perforation  No  Before surgery  During surgery  After surgery | 99 (92.5)  4 (3.7)  1 (0.9)  3 (2.8) | 48 (90.6)  2 (3.8)  1 (1.9)  2 (3.8) | 51 (94.4)  2 (3.7)  -  1 (1.9) | 0.70 |
| Tumour spill | 4 (3.7) | 3 (5.7) | 1 (1.9) | 0.30 |
| Adjuvant chemotherapy | 54 (50.5) | 0 (0.0) | 54 (100.0) | **<0.001** |
| Recurrence | 43 (40.2) | 18 (34.0) | 25 (46.3) | 0.19 |
| CRC mortality | 35 (32.7) | 15 (28.3) | 20 (37.0) | 0.34 |
| Overall mortality | 56 (52.3) | 30 (56.6) | 26 (48.1) | 0.38 |
| Follow up, mean (s.d.) in months | 60.8 (32.0) | 65.0 (35.1) | 56.8 (28.6) | 0.20 |

**Supplementary Table 2**Association between MVD and TSP (continuous data), and expression of HIF1A and VEGFA (dichotomized data) for both stage II and stage III colon cancer, using Spearmann’s rho correlation analysis (^*^ = p < 0.050).

|  | **Stage II** | | **Stage III** | |
| --- | --- | --- | --- | --- |
| Association of MVD with: | Spearman’s rho | P-value | Spearman’s rho | P-value |
| TSP | 0.059 | 0.70 | 0.144 | 0.30 |
| HIF1A | 0.091 | 0.53 | -0.119 | 0.40 |
| VEGFA | **-0.331** | **0.020^*^** | -0.013 | 0.93 |
