# Supplementary material for: Prognostic value of microvessel density in stage II and III colon cancer patients: a retrospective cohort study
Source: BMC Gastroenterol. 2019 Aug 16;19:146. doi: 10.1186/s12876-019-1063-4 (PMC6698008; doi:10.1186/s12876-019-1063-4)
Supplement: Supplementary file 3 — Figure S1. DFS stratified for high and low stromal percentage. Figure S2. DFS stratified for high and low expression of HIF1A. Figure S3. DFS stratified for high and low expression of VEGFA. (PPTX 101 kb) [file 12876_2019_1063_MOESM3_ESM.pptx]

## Slide 1
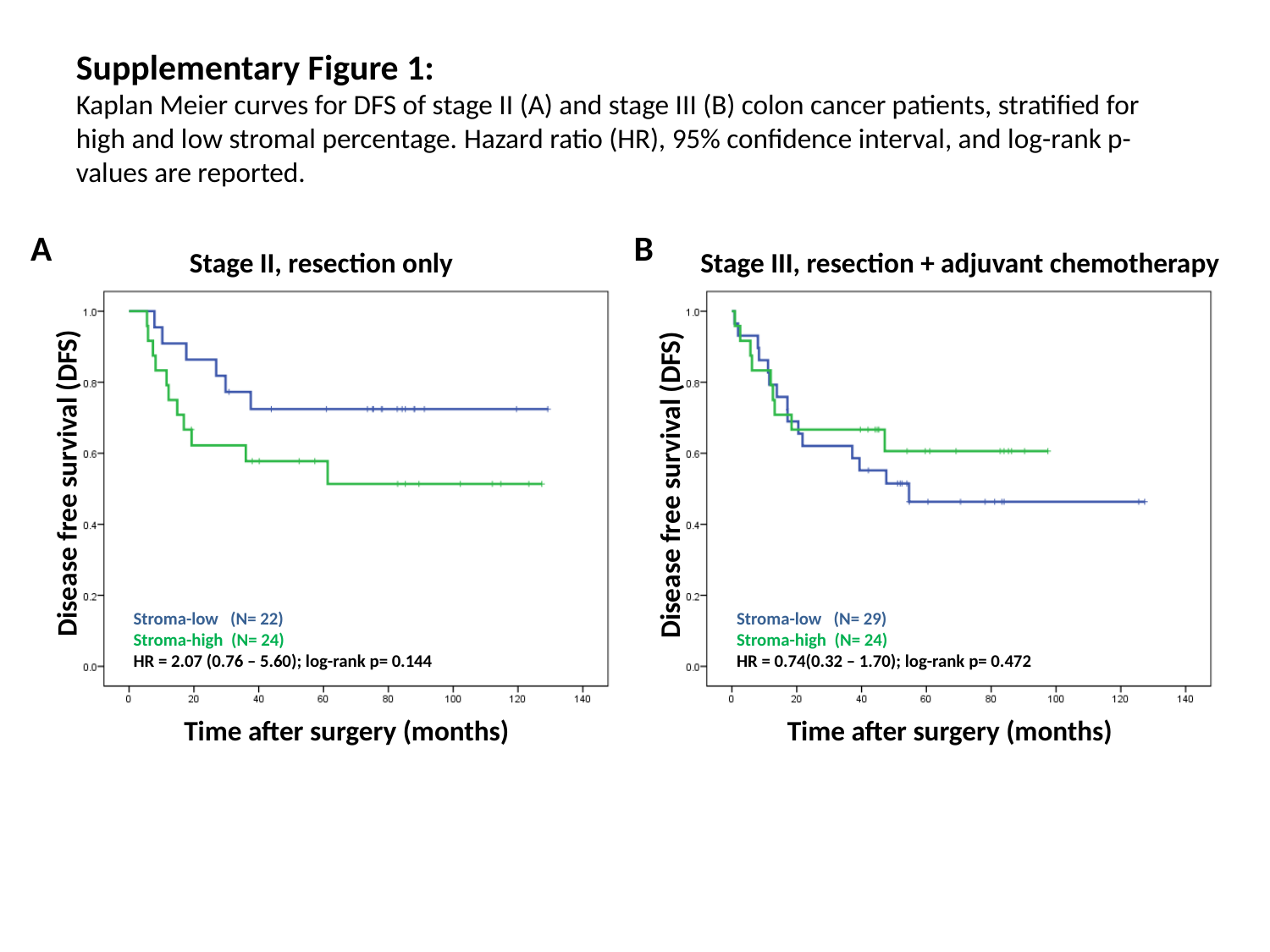

Supplementary Figure 1:
Kaplan Meier curves for DFS of stage II (A) and stage III (B) colon cancer patients, stratified for high and low stromal percentage. Hazard ratio (HR), 95% confidence interval, and log-rank p-values are reported.
A
B
Stage II, resection only
Stage III, resection + adjuvant chemotherapy
Disease free survival (DFS)
Stroma-low (N= 22)
Stroma-high (N= 24)
HR = 2.07 (0.76 – 5.60); log-rank p= 0.144
Time after surgery (months)
Disease free survival (DFS)
Stroma-low (N= 29)
Stroma-high (N= 24)
HR = 0.74(0.32 – 1.70); log-rank p= 0.472
Time after surgery (months)

## Slide 2
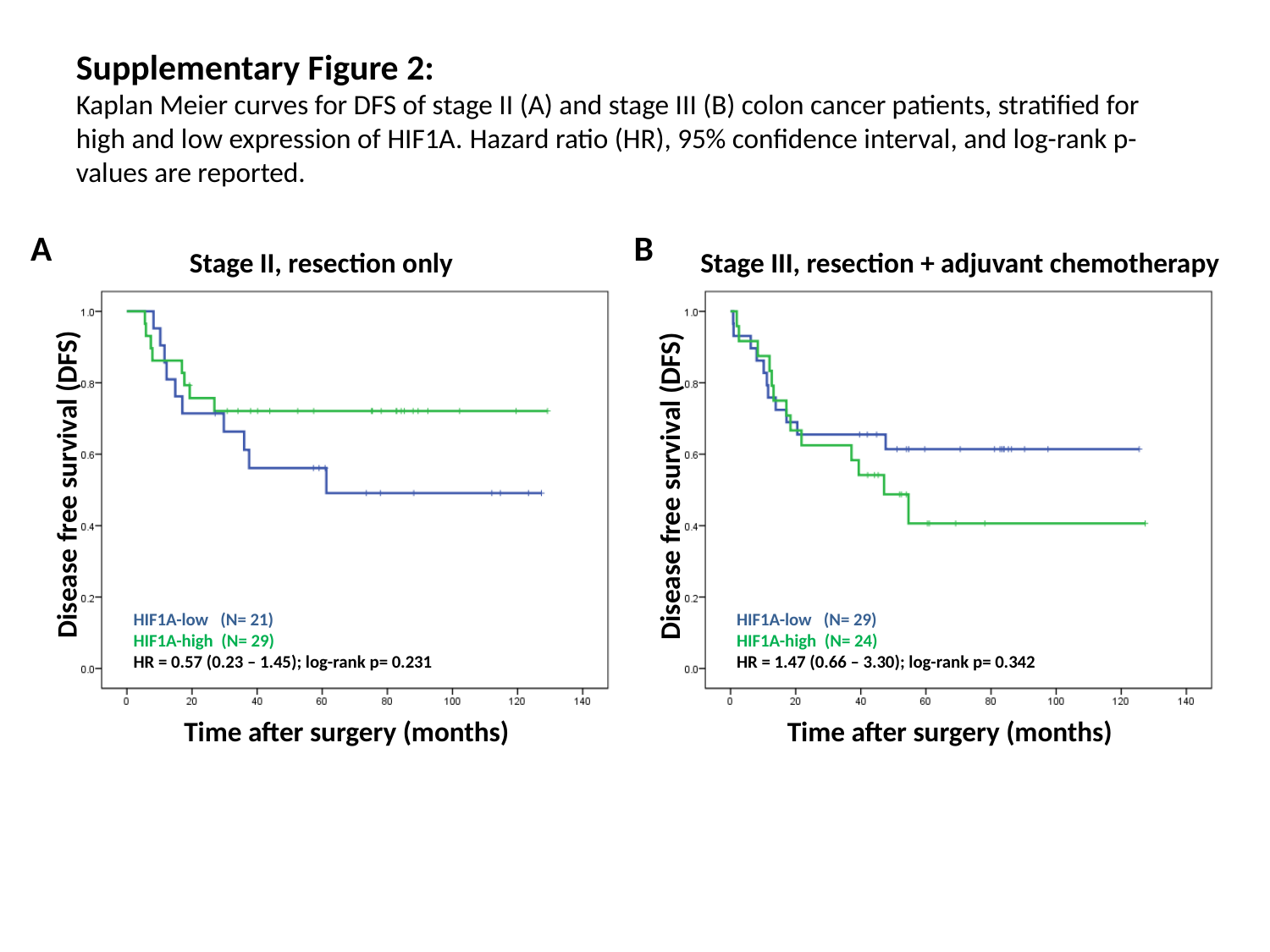

Supplementary Figure 2:
Kaplan Meier curves for DFS of stage II (A) and stage III (B) colon cancer patients, stratified for high and low expression of HIF1A. Hazard ratio (HR), 95% confidence interval, and log-rank p-values are reported.
A
B
Stage II, resection only
Stage III, resection + adjuvant chemotherapy
Disease free survival (DFS)
Disease free survival (DFS)
HIF1A-low (N= 21)
HIF1A-high (N= 29)
HR = 0.57 (0.23 – 1.45); log-rank p= 0.231
HIF1A-low (N= 29)
HIF1A-high (N= 24)
HR = 1.47 (0.66 – 3.30); log-rank p= 0.342
Time after surgery (months)
Time after surgery (months)

## Slide 3
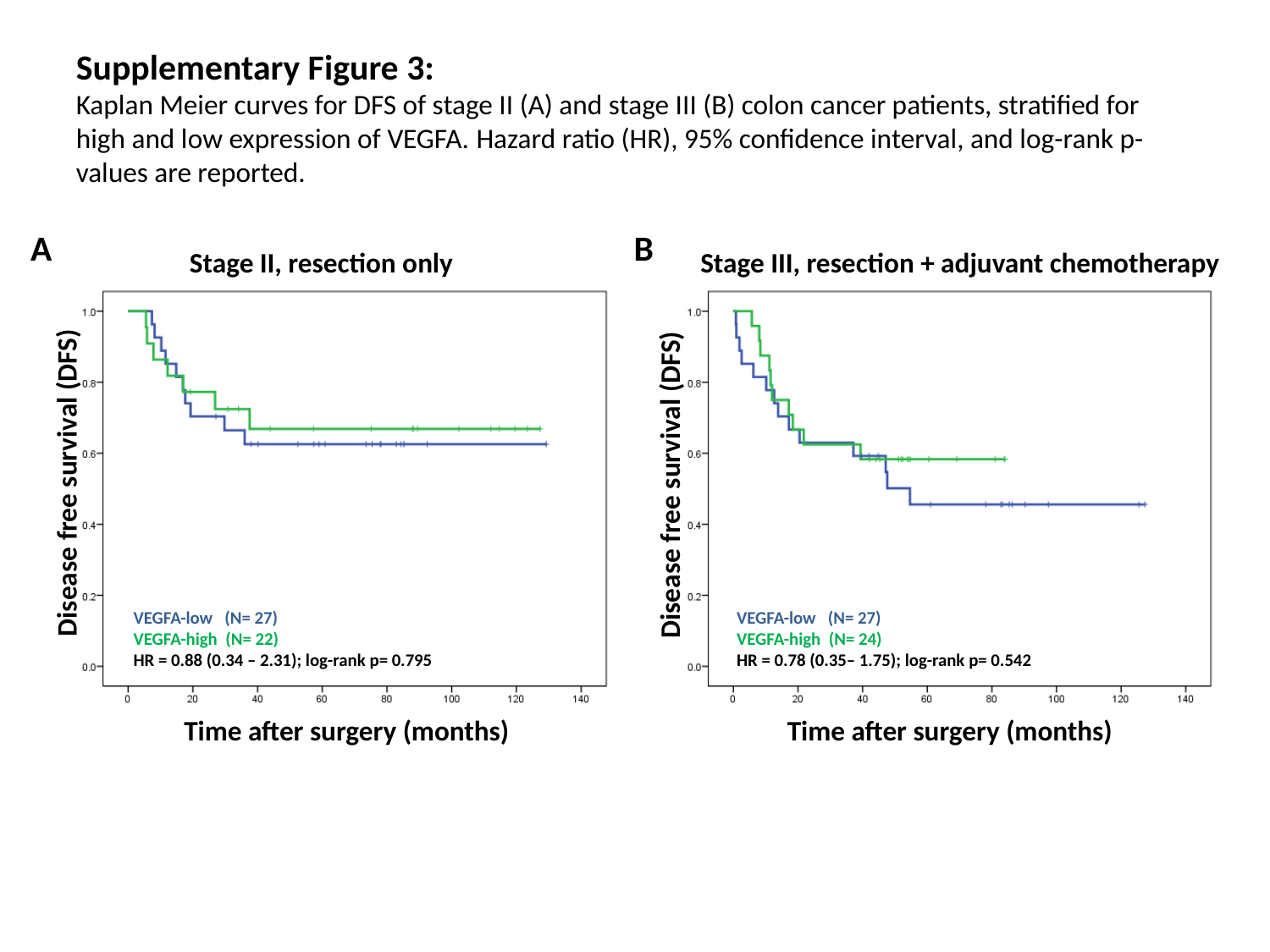

Supplementary Figure 3:
Kaplan Meier curves for DFS of stage II (A) and stage III (B) colon cancer patients, stratified for high and low expression of VEGFA. Hazard ratio (HR), 95% confidence interval, and log-rank p-values are reported.
A
B
Stage II, resection only
Stage III, resection + adjuvant chemotherapy
Disease free survival (DFS)
Disease free survival (DFS)
VEGFA-low (N= 27)
VEGFA-high (N= 22)
HR = 0.88 (0.34 – 2.31); log-rank p= 0.795
VEGFA-low (N= 27)
VEGFA-high (N= 24)
HR = 0.78 (0.35– 1.75); log-rank p= 0.542
Time after surgery (months)
Time after surgery (months)
